# Gut Microbiota Are Associated With Psychological Stress-Induced Defections in Intestinal and Blood–Brain Barriers

**DOI:** 10.3389/fmicb.2019.03067

**Published:** 2020-01-15

**Authors:** Shaohui Geng, Liping Yang, Feng Cheng, Zhumou Zhang, Jiangbo Li, Wenbo Liu, Yujie Li, Yukun Chen, Yu Bao, Lin Chen, Zihao Fei, Xinmin Li, Junlin Hou, Yuan Lin, Zhilin Liu, Shuai Zhang, Hengtao Wang, Qing Zhang, Honggang Wang, Xiaodan Wang, Jingtao Zhang

**Affiliations:** ^1^First Clinical Medical College, Henan University of Chinese Medicine, Zhengzhou, China; ^2^Basic Medical College, Henan University of Chinese Medicine, Zhengzhou, China; ^3^Rehabilitation Medical College, Henan University of Chinese Medicine, Zhengzhou, China; ^4^School of Pharmacy, Henan University of Chinese Medicine, Zhengzhou, China; ^5^Acupuncture and Massage College, Henan University of Chinese Medicine, Zhengzhou, China

**Keywords:** gut microbiota, dysbiosis, psychological stress, intestinal barrier, blood–brain barrier, tight junction, communication box

## Abstract

Altered gut microbiota has been identified during psychological stress, which causes severe health issues worldwide. The integrity of the intestinal barrier and blood–brain barrier regulates the process of bacterial translocation and can supply the nervous system with real-time information about the environment. However, the association of gut microbiota with psychological stress remains to be fully interpreted. In this study, we established a psychological stress model using an improved communication box and compared the expression of tight junction proteins in multiple regions of the intestinal (duodenum, jejunum, ileum) and blood–brain (amygdala, hippocampus) barriers between model and control rats. We also conducted fecal microbiota analysis using 16S rRNA gene sequencing. Expression levels of the stress-related indicators adrenocorticotropic hormone, NR3C1,2, and norepinephrine were increased in the model group compared to control group. Psychological stress reduced brain and intestinal levels of tight junction proteins, including claudin5, occludin, α-actin, and ZO-1. Microbiota analysis revealed elevated microbial diversity and fecal proportions of *Intestinimonas*, *Catenisphaera*, and *Globicatella* in the model group. Further analysis indicated a negative correlation of *Allisonella* and *Odoribacter*, as well as a positive correlation of *norank_f__Peptococcaceae*, *Clostridium_sensu_stricto_1*, and *Coprococcus_2*, with claudin5, occludin, α-actin, and ZO-1. Our use of a rodent model to explore the association between compromised intestinal and blood–brain barriers and altered fecal microbiota under psychological stress improves our understanding of the gut–brain axis. Here, cues converge to control basic developmental processes in the intestine and brain such as barrier function. This study provides new directions for investigating the pathogenesis of emotional disorders and the formulation of clinical treatment.

## Introduction

Stressful life events play an important role in the occurrence of mental illness; however, the unclear pathogenesis leads to many obstacles when forming a treatment plan (Goyal et al., 2014). The human intestine, called “the second brain”, harbors nearly 100 trillion types of bacteria (Partrick et al., 2018), and there is compelling evidence for bidirectional interaction between stress and the microbiome (Picard and McEwen, 2018). The specific communication mechanisms, however, remain to be studied.

The integrity of the intestinal barrier and blood–brain barrier (BBB) is necessary for protecting the body from external stimulation and disturbance of the internal environment. Repeated exposure to social stress can alter the diversity and composition of gut microbiota, accompanied by changes in microbial metabolites, cytokines, chemokines, and monoamine transmitters, which regulate behavior by stimulating the peripheral and central nervous systems (Braniste et al., 2014; Stilling et al., 2016). For instance, when these two essential barriers are damaged, short-chain fatty acids, lipopolysaccharide, and IL-6 can pass through the intestinal epithelium and increase their circulatory concentrations (Frost et al., 2014; Zuo et al., 2019). Subsequently, these products are transported through the defective BBB and enter into the brain through a saturated transport mechanism, giving rise to abnormal emotions (Fiorentino et al., 2016; Rahman et al., 2018). There are no systematic reports illustrating the effects of psychological stress on different tight junction proteins and regions of the intestinal barrier and BBB; however, different regions are associated with different functions and physiological environments.

Modeling of psychological stress generally includes the simultaneous presence of physiological and psychological stressors, as in the chronic unpredictable mild stress (CUMS) (Zhu et al., 2017; Sun et al., 2018), social disruption stressor (Bharwani et al., 2016), and social conflict stress (Partrick et al., 2018) models, while psychological and physical stress differentially influence cognitive, emotional, and physical function of animals (Kavushansky et al., 2009; Liu et al., 2018). The clinical occurrence of emotional disorders is primarily related to psychological stress factors, yet the existing psychological stress models are largely mixed with physiological stimuli, such as electric shock, cold, and tussle. This is not conducive to revealing the relationship between psychological stress and gut microbiota; thus, a real psychological stress model should be established.

In this study, we established a real psychological stress model based on that of Gomita and Ramsey (Gomita et al., 1983; Ramsey and Van Ree, 1993) by employing a specially designed communication box patented by our team (Liping et al., 2012) and used for research on real psychological stress (Junlin et al., 2018; Liping et al., 2018). We then evaluated this rat model from the perspectives of developmental and emotional phenotypes, monoamine neurotransmitters, and glucocorticoid receptors, and analyzed four tight junction proteins in the duodenum, jejunum, ileum, amygdala, and hippocampus. Furthermore, fecal microbiota analysis was conducted using 16S rRNA gene sequencing. We are committed to exploring the association between compromised intestinal and blood–brain barriers and altered fecal microbiota under psychological stress stimulation.

## Materials and Methods

### Animal Care

Four-week-old, female, specific pathogen free SD rats were purchased from Vital River Laboratory Animal Technology Company (Beijing, China) and housed in the laboratory animal center of the Henan University of Chinese Medicine (Henan, China). The rats were housed in sterile animal colonies under 25°C ± 5°C, 65% ± 5% humidity, and 12 h light-dark cycle. This study was conducted at Henan University of Chinese Medicine under the guidelines of the National Institutes of Health Guide for the Care and Use of Laboratory Animals approved by the Animal Ethics Committee of Henan University of Chinese Medicine (Permit Number: DWLL2018030017).

### Experimental Protocol

After 7 days of accommodation, a baseline test of rats was carried out through an open field experiment. Thirty rats with consistent emotional level were randomly divided into two groups (control group and psychological-stress model group, *n* = 15 each). Rats in the model group received psychological-stress stimulation through the communication box system for 28 days (Tang et al., 2017; Junlin et al., 2018). During modeling, rats in the control and model groups were fed regularly, and the consumption of water and food in both groups was monitored daily. The weight of rats in each group was measured on the 7th, 14th, 21st, and 28th day. After modeling, feces were collected from six rats (Javurek et al., 2016; Zheng et al., 2017) in each group and stored at −80°C for examination. The emotions of the rats were evaluated through the open field test using the following procedures. (i) Blood was collected from eyeballs of 12 rats (*n* = 6, each group) for serum adrenocorticotropic hormone (ACTH) analysis using an ELISA kit; these rats were then sacrificed for isolating the cortex, amygdala, and hippocampus on ice. (ii) High-performance liquid chromatography (HPLC) was used to determine norepinephrine (NE) content in cortex, amygdala, and hippocampus. Subsequently, another ten (*n* = 5, each group) rats were perfused with polyformaldehyde for rapid intestine and brain extraction. (iii) Brains were immersed in polyformaldehyde fixative and cut into parts to observe the expression of NR3C1 and NR3C2 in the cortex by immunohistochemistry. (iv) The remaining brain parts and intestines were immersed in polyformaldehyde fixative to observe the expression of claudin5, occludin, α-actin, and ZO-1 by immunohistochemistry, and to observe the structure of tight junctions by electron microscopy.

### Equipment and Method for Preparing Psychological Stress Model

**FIGURE 1:**
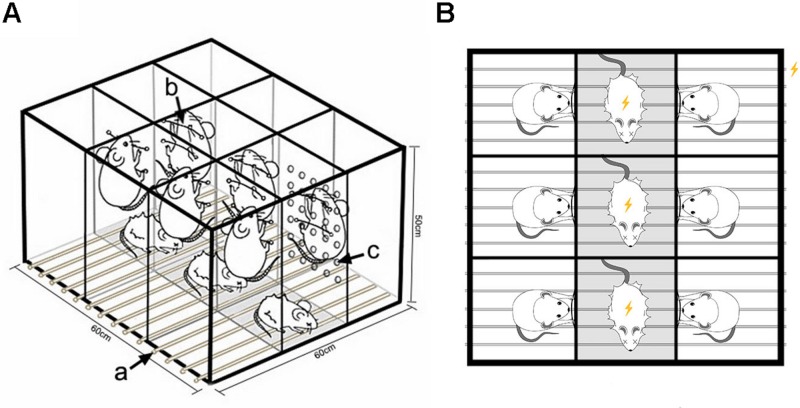
Equipment for the psychological stress rodent model. **(A)** Schematic diagram for construction of the modeling device. **(a)** Wires connected to a small animal stimulator can provide plantar electrical stimulation. **(b)** Wires fixed at the separator in the direction of rats getting an electric shock can be grasped by model rats to escape electric shock stimulation. **(c)** Thirty holes with diameter of 1 cm are evenly distributed to facilitate capturing the fear signals of model rats given electric shock. **(B)** Experimental design. Rats in the middle row received electrical stimulation, while rats in the two adjacent rows received mental stimulation by watching, listening, and smelling the rats given electric shock.

The communication box system (Gomita et al., 1983; Ramsey and Van Ree, 1993) was improved and patented by our team (Liping et al., 2012). In the interior of the box, transparent partitions with 30 uniformly distributed holes of 1 cm in diameter divide the space into nine small chambers of 20 cm× 20 cm× 50 cm in size (Figure 1). At the bottom of the box is an electric shock plate consisting of a uniformly arranged stainless steel wire, which can be connected to an electrical stimulator to trigger electrical stimulation. Extra rats to be given electric shock were placed into the three chambers of the middle row, whereas model rats were randomly placed into the other six chambers. In each of the six compartments where the model rats were placed, two wires were fixed at a height of about 25 and 35 cm from the bottom in the direction of rats being shocked. The model rats, after training, could grip the wires to avoid the electric shock. Fear signals were evoked in the model rats by listening to the screeches, watching the jumping, and smelling the odor of the rats being given an electric shock, along with the stimulation of a fire alarm.

At the end of adaptive feeding, the psychological stress model was conducted for half an hour per day for 28 days. The details are as follows: (i) At 8:30 am, the rats to be given electric shock and the model rats were placed in the improved communication box chamber, respectively. (ii) Using a stopwatch for timing, the equipment was electrified every other 6 s to give a momentary stimulation (20 mA, 5 ms), along with a fire alarm. Modeling lasted for 60 s, followed by a 60-s interval. The operation was conducted 15 times in accordance with the above method, totaling 30 min.

### Open Field Test

After the psychological stress stimulation lasting for 28 days, an open field test was employed to evaluate rat behavior. All procedures were conducted according to previous research (Zhu et al., 2017). The open field was an arena (100 cm × 100 cm × 50 cm) with black sides and bottom, divided into 25 equal-sized squares by white lines. After 1 h of adaptation in the testing room, rats were placed individually into the device. The operators observed the behavior of each rat for 3 min through a video camera. The number of bottom squares passed by rats was recorded as horizontal motion, while the number of times the rats lifted their forelimbs was recorded as vertical motion. To eliminate odor interference, equipment was wiped with alcohol gauze before each test.

### Feces Sample Collection and Microbiome Determination

After modeling, feces were collected from twelve rats (*n* = 6, each group) in a super-clean workbench using the following steps: (i) the workbench was sterilized using ultraviolet radiation for 15 min; (ii) one person held the rat and massaged its abdomen to promote excretion of feces. Another person wearing sterile gloves collected the feces using sterile filter paper; (iii) the feces were transferred to a sterile centrifuge tube and stored at −80°C for microbiome determination.

DNA purification, 16S rRNA gene amplification, and Illumina MiSeq sequencing were performed by Shenzhen Microeco Technology Ltd., (Shenzhen, China). Total DNA was extracted from feces using an OMEGA-soil DNA kit. PCR amplification was carried out using the V3-V4 variable region sequence of the 16S rRNA gene as target and 338F-806R with barcode sequences as primers. PCR products were sequenced on the Illumina MiSeq PE300 platform to obtain V3-V4 variable region base sequence information of bacterial 16S rRNA genes. Sequencing fragments were clustered by operational taxonomic unit (OTU) using the QIIME2.0 software package. Representative sequences of each OTU were compared with sequences in the Silva database to identify the OTUs and determine their corresponding abundance information. Chao, Sobs, Ace, bootstrap, and jackknife indexes were calculated to evaluate the richness and uniformity of bacterial flora in samples. PCA analysis, LEfSe analysis, and ANOVA were used to identify the characteristic bacteria in each group. A Spearman correlation heatmap was used to analyze the correlation between bacterial abundance and tight junction protein content.

### Enzyme-Linked Immunosorbent Assay

The level of ACTH in serum was determined using an ELISA kit. All procedures were conducted following the manufacturer’s instructions: (i) the absorbance of each sample was detected at 450 nm wavelength; (ii) the absorbance was taken as the ordinate, and the corresponding standard concentration was taken as the abscissa to draw a standard curve; (iii) the concentration of ACTH in each sample was calculated using the regression equation of the standard curve. All serum samples were analyzed in triplicate.

### Immunohistochemistry and HPLC

Expression levels of NR3C1 and NR3C2 in the cortex were detected by immunohistochemistry. Norepinephrine (NE) levels in the brain (amygdala, hippocampus, and cortex) were detected by HPLC. To eliminate interference from the researchers and ensure the accuracy of the experimental results, we entrusted the immunohistochemical detection to Zhengzhou Dianjie Technology Co., Ltd., and entrusted the determination of NE in the brain to the Medical Laboratory Center of Chinese Academy of Traditional Chinese Medicine.

### Electron Microscopy

Transmission electron microscope images were prepared by the Electron Microscope Center of Scientific Research and Experiment Center of Henan University of Traditional Chinese Medicine. The samples were processed as follows: (i) tissues less than 1 mm^3^ were immobilized with 2.5% glutaraldehyde for 4 h and washed four times with 0.1 mol PBS for 15 min each time; (ii) after rinsing, tissues were placed into 1% osmium acid fixative solution and fixed again for 1.5 h; (iii) tissues were then rinsed four times with 0.1 mol PBS, for 15 min each time. Tissue blocks were fixed twice in 50, 70, 80, and 100% ethanol solutions and twice in 100% acetone, each for 15 min. The dehydrated tissue blocks were embedded in a mixture of epoxy resin 812 and acetone (1:1), epoxy resin 812 and acetone (2:1), and pure epoxy resin 812, respectively, and placed overnight at room temperature; (iiii) after the tissue blocks were polymerized, they were cut into 50–60 nm slices using an ultra-thin slicing machine and dyed in saturated uranium dioxide acetate solution for 20 min. After rinsing and drying, the sections were observed and photographed by transmission electron microscopy.

### Statistical Analysis

Results are presented as mean ±SD. Statistical analysis was performed by independent sample *t*-test, using IBM SPSS Statistics 22. *P*-value < 0.05 was considered statistically significant. 16S rRNA gene data were analyzed on the free online Majorbio I-Sanger Cloud Platform^1^. Sequencing data have been deposited under the number SRP201262 or PRJNA548591 in the NCBI database.

## Results

### Assessment of the Psychological Stress Model

**FIGURE 2:**
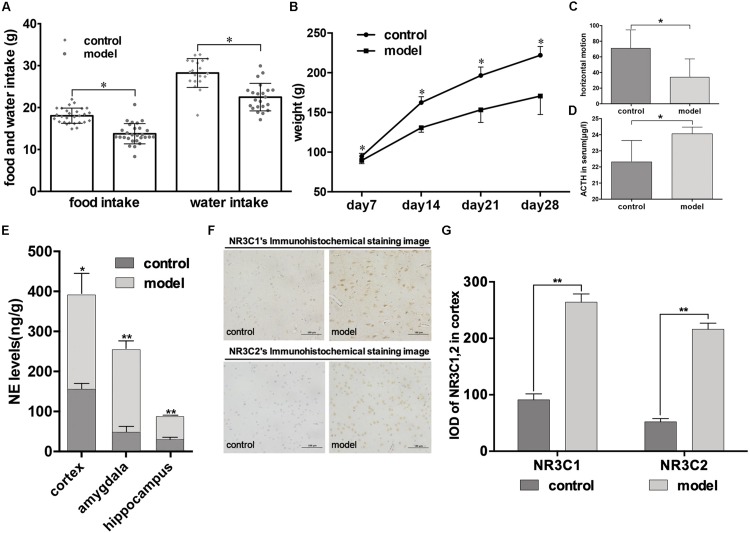
Impact of psychological stress stimulation on emotional phenotypes and indicators in rats. **(A,B)** Development of phenotypic changes after psychological stress. **(A)** Daily food and water intake in control and model groups (lozenges: control; circles: model; monitoring time: 28 days). **(B)** Weekly weight of rats in control and model groups (circles: control; squares: model; monitoring time: per week after stimulation, for 4 weeks). **(C,D)** Emotional phenotype and ACTH level changes after modeling (light gray: control; dark gray: model). **(C)** Results of the open field test were measured after stimulation for 28 days. Horizontal motion of rats in the open field was calculated (control, *n* = 12; model, *n* = 12). **(D)** ACTH levels in both groups were recorded (*n* = 6, each group). **(E)** NE levels in cortex, amygdala, and hippocampus measured by HPLC (*n* = 6, each group). **(F,G)** Immunohistochemical analysis of NR3C1 and NR3C2 expression in cortex on day 28 (*n* = 5, each group). Data shown as mean ± SD; ^∗^*P* < 0.05, ^∗∗^*P* < 0.01, model group vs. control group.

From continuous monitoring, we found that psychological stress caused negative effects on rat development, reflected by rats in the model group consuming less food and water, and losing significant weight (*P* < 0.05, Figures 2A,B). The open field test revealed the same trend, with rats in the model group exhibiting less horizontal motion (*P* < 0.05, Figure 2C). After 28 days’ stimulation, there was a significant increase in serum ACTH levels in the model group compared with the control group as detected by ELISA (*P* < 0.05, Figure 2D). The presence of the neurotransmitter NE in the brain is an essential indicator of psychological stress, and our results showed that psychological stress elevated NE levels in different brain areas (cortex, amygdala, and hippocampus) of model rats compared to rats in the control group, as measured by HPLC (cortex: *P* < 0.05; amygdala, hippocampus: *P* < 0.01; Figure 2E). Moreover, different expression levels of the glucocorticoid receptor proteins NR3C1 and NR3C2 in the cortex were found between the two groups, with higher NR3C1 and NR3C2 expression levels in the cortex of model rats (*P* < 0.01, Figure 2G). The immunohistochemical staining images are shown in Figure 2F: strong positive staining in the model group, and mildly positive staining in the control group.

### Psychological Stress Decreased the Expression of Tight Junction Proteins in Intestinal and Blood–Brain Barriers

**FIGURE 3:**
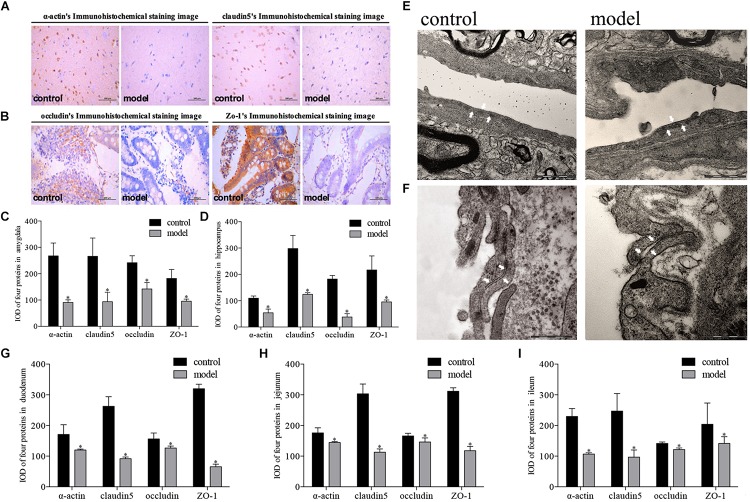
Impact of psychological-stress stimulation on tight junction proteins in intestinal and blood–brain barriers. **(A,C–E)** Decreased expression of tight junction proteins in the amygdala and hippocampus. **(A)** Representative immunohistochemical staining of α-actin and claudin5 in the amygdala (model and control markers are located in the lower right corner of the image). **(C)** IOD values for expression levels of four proteins in the amygdala. **(D)** IOD values for expression levels of four proteins in the hippocampus (light gray: control; dark gray: model; *n* = 5, each group). **(E)** Representative electron microscopy pictures of tight junctions of the BBB in the amygdala (tight junction indicated by white arrow; *n* = 3, each group). **(B,F–I)** Decreased expression of tight junction proteins in duodenum, jejunum, and ileum. **(B)** Representative immunohistochemical staining of occludin and ZO-1 in the duodenum (model and control markers are located in the lower right corner of the image). **(F)** Representative electron microscopy pictures of tight junctions of the intestinal barrier in the duodenum (tight junction indicated by white arrow; *n* = 3, each group). **(G)** IOD values for expression levels of four proteins in the duodenum. **(H)** IOD values for expression levels of four proteins in the jejunum. **(I)** IOD values for expression levels of four proteins in the ileum (light gray: control; dark gray: model; *n* = 5, each group). Data shown as mean ± SD; ^∗^*P* < 0.01, model group vs. control group.

Modeling revealed that real psychological stress could reduce the expression of tight junction proteins in multiple areas of the BBB (amygdala, hippocampus) and intestinal barrier (duodenum, jejunum, and ileum). Significant differences in expression levels of the four proteins in these areas were observed between the two groups of rats. Reduced expression of the four proteins was found in both the brain and the intestine in the model group. We observed strongly positive immunohistochemical staining in the control group and mildly positive staining in the model group (Figures 3A,B, representative images from amygdala and duodenum). *Post hoc* tests revealed that the IOD values of immune-positive signals for the four proteins in the model group were lower than those in the control group both in brain and intestine (*P* < 0.01, Figures 3C,D,G,H,I). Meanwhile, similar morphological results were also observed by electron microscopy: the tight junction between the vascular endothelium of the BBB in the model group was looser than that in the control group, and the basement membrane was broken (Figures 3E,F).

### Imbalanced Gut Microbiota in Rats Under Psychological Stress

**FIGURE 4:**
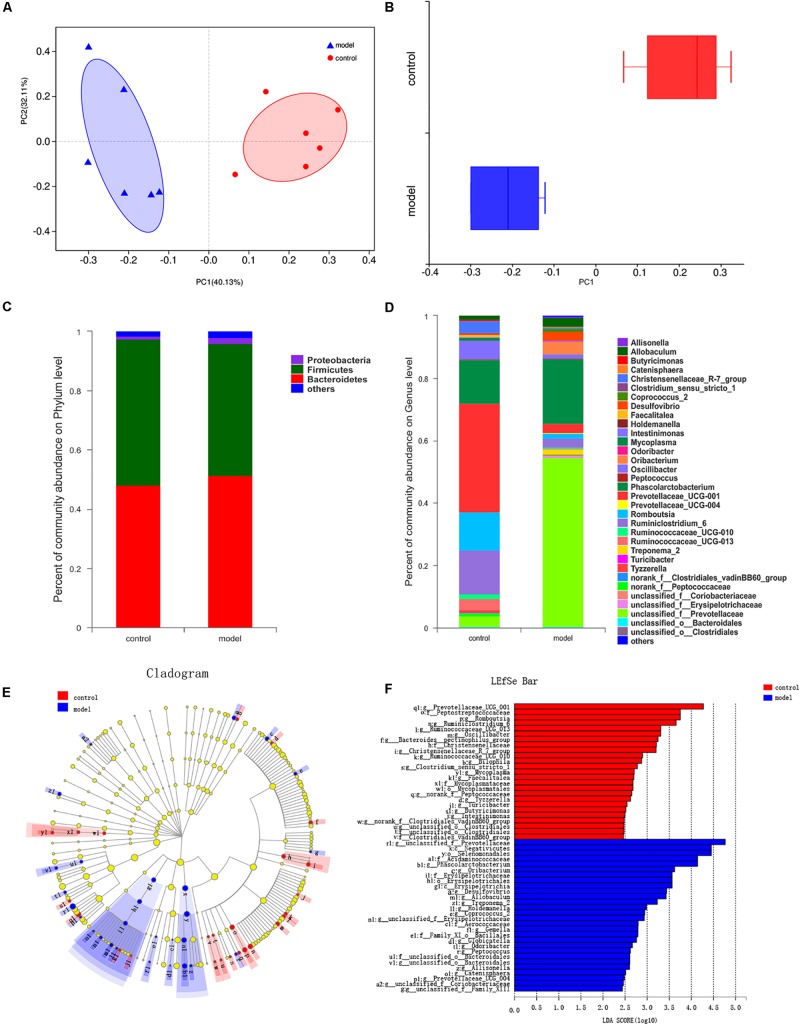
Effects of psychological-stress stimulation on gut microbiota composition. **(A)** Plots shown were generated using the abund_jaccard-based PCoA (*R*^2^ = 0.4058, *P* < 0.01). The *R*^2^ value was calculated by Adonis algorithm. **(B)** The discrete distribution of different groups of samples on PC1 axis. **(C)** Community abundance in gut microbiota at the phylum level. **(D)** Differences in gut microbiota composition at the genus level. **(E)** Results of LEfSe analysis. Nodes with different colors represent microbial groups that are significantly enriched in the corresponding rat groups and have significant effects on the differences between groups; pale yellow nodes represent microbial groups that show no significant differences or no significant effects on the differences between groups. **(F)** Key taxa found by LDA analysis (multigroup comparison strategy: one-against-all, LDA > 2, *P* < 0.05). The higher the LDA score, the greater the impact of the representative species abundance on the difference between groups.

We generated 639245 high-quality V3-V4 region 16S rRNA gene sequences from 12 samples, representing 13 phyla, 23 classes, 32 orders, 53 families, 165 genera, and 897 OTUs. First, we examined alterations in the microbial composition with the principal coordinate analysis (PCoA) clustering. PC1 accounted for 40.13% of the variation and PC2 accounted for 32.11%. PCoA results displayed an obvious clustering of microbiota composition for two groups (Figure 4A, *R*^2^ = 0.4058, *P* < 0.01). In addition, we can get the consistency information of samples from the box graph of sample discrete distribution on PC1 axis (Figure 4B). The composition of gut microbiota in rats was significantly modulated after psychological stress. At the phylum level, the most abundant bacteria were Bacteroidetes (51.43% of reads in model group, 48.12% in control group), Firmicutes (44.51% in model group, 49.38% in control group), and Proteobacteria (1.96% in model group, 1.08% in control group). These three phyla constituted 98.24% of the total microbiota (Figure 4C). The percentage composition in the two rat groups at the phylum level was similar, and there was no marked difference between the control and model groups (*P* > 0.05).

The impact of stimulation on the structure of the gut microbiota was clear at the genus level. We identified 36 genera showing a significant difference in abundance between the control group and the model group (Figure 4D). Eight genera or genus-level groups were only present in the model group (*Allisonella*, *Globicatella*, *Holdemanella*, *Odoribacter*, *Prevotellaceae_UCG-004*, *Treponema_2*, *unclassified_f__Family_ XIII*, and *unclassified_f_Prevotellaceae*), whereas *Clostridium_ sensu_stricto_1* was only found in the control group. Meanwhile, increased abundance of 19 genera (including *unclassified_ f__Prevotellaceae* and *Phascolarctobacterium*) and decreased abundance of 17 genera (including *Prevotellaceae_UCG-001*, *Ruminiclostridium_6*, and *Romboutsia*) was detected in the model group when compared with the control group (*P* < 0.05).

According to the LEfSe and LDA analysis, 53 taxa distinguished the two groups: 25 for the control group and 28 for the model group (Figures 4E,F). There was no biomarker detected in the two groups at the phylum level. At the class level, Negativicutes and Erysipelotrichia played an essential role in the model group. At the genus level, *Phascolarctobacterium*, *Oribacterium*, *Desulfovibrio*, *Allobaculum*, and *Treponema_2* characterized the model group, while *Prevotellaceae_UCG_001*, *Romboutsia*, *Ruminiclostridium_6*, *Ruminococcaceae_UCG_013*, *Oscillibacter*, *Bacteroides__pectinophilus_group*, and *Christensen- ellaceae_R_7_group* were important in the control group. Inte- restingly, there were 11 stress-related genera: *Romboutsia*, *Desulfovibrio*, *Ruminococcaceae_UCG-010*, *Treponema_2*, *Tyzze- rella*, *unclassified_o__Bacteroidales*, *Intestinimonas*, *Clostridium_ sensu_stricto_1*, *Catenisphaera*, *Gemella*, and *Globicatella*.

Meanwhile, at the genus level, the α-diversity of Chao, Sobs, Ace, bootstrap, and jackknife indexes in the model group, which represent community richness, were higher than those of the control group (Supplementary Table S1, *P* < 0.05). There was also a marked difference between the two groups in the qstat index, which reflects community diversity (Supplementary Table S1, *P* < 0.05). These substantial pieces of evidence indicated that psychological-stress stimulation had a significant impact on the gut microbiome: it changed the structure of the gut microbiome and increased the community richness and diversity.

### Correlation Between Intestinal/Blood–Brain Tight Junction Proteins and Gut Microbiota Dysbiosis

**FIGURE 5:**
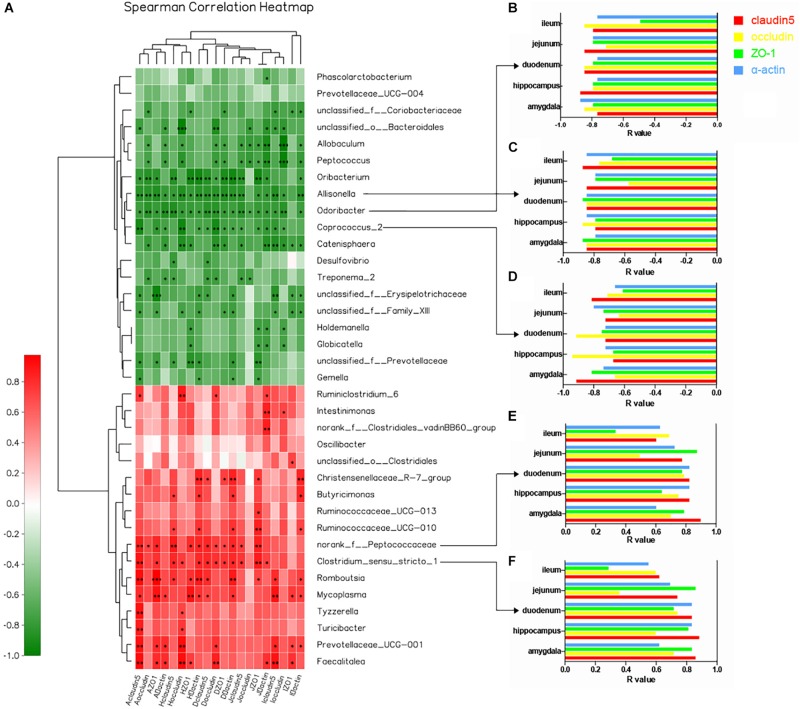
Correlation between core bacteria and tight junction proteins in multiple regions of the intestinal barrier and BBB at the genus level. **(A)** Heatmap correlation analysis of brain and intestinal tight junction proteins and core gut microbiota at the genus level. The *x*-axis represents tight junction proteins in different regions of the brain and intestine. The *y*-axis represents species at the genus level. *R*- and *P*-values were obtained by calculation using the Spearman Grade Coefficient. The *R*-value is shown in different colors: red, positive correlation; green, negative correlation. The legend on the right shows the color intervals of different *R*-values; depth of color indicates degree of correlation. ^∗^*P* < 0.05. Cluster trees representing species and environmental factors (left and upper) are shown. ^∗^0.01 < *P* < 0.05, ^∗∗^0.001 < *P* < 0.01, ^∗∗∗^*P* < 0.001. **(B–F)** *R*-value histograms showing correlation of five representative genera and tight junction proteins at different sites. The *y*-axis represents different regions of the brain and intestine. The *x*-axis represents specific *R*-values between the flora and four tight junction proteins. Opening of the histogram to the left means that the *R*-value is negative, and there is a negative correlation. Otherwise, there is a positive correlation (red: claudin5; yellow: occludin; green: ZO-1; blue: α-actin).

We used heatmap correlation analysis to calculate the correlation between brain and intestinal tight junction proteins and the core bacteria identified from the LDA results. Interestingly, the four tight junction proteins (claudin5, occludin, α-actin, and ZO-1) in the duodenum, jejunum, and ileum showed strong relationships with the key gut microbiota at the genus level, which distinguished the two groups. It is notable that the similar results were detected in the BBB including amygdala and hippocampus. The *R* value between the tight junction proteins and gut microbiota which revealed the strength of this connection was represented in the form of the heatmap (Figure 5A). Particularly, five genera showed notable correlation with tight junction proteins; two positive correlations: *norank_f_Peptococcaceae* (Figure 5E) and *Clostridium_sensu_stricto_1* (Figure 5F), and three negative correlations: *Allisonella* (Figure 5B), *Odoribacter* (Figure 5C), and *Coprococcus_2* (Figure 5D).

## Discussion

In this study, we explored the association of defective intestinal and blood–brain barriers with altered fecal microbiota under psychological stress, to improve our understanding of the gut–brain axis. We found that real psychological stress could lead to impaired intestinal and blood–brain barriers, characterized by decreased expression of four tight junction proteins, looser tight junctions, and broken basement membrane. Moreover, there was a notable correlation between disordered microbial composition and compromised intestinal and blood–brain barriers.

### Assessment of Improved Psychological Stress Model

Previous psychological stress modeling methods [e.g., CUMS (Sun et al., 2018), social disruption stressor (Bharwani et al., 2016), and social conflict stress (Partrick et al., 2018) models] have included mixtures of psychological and physiological stimulation; however, diversed stimulation modes have different effects on animals, including psychological, behavioral, learning, memory, neuroendocrine, and neurochemical (Katsura et al., 2002; Haleem et al., 2014). Thus, in this study, we established a real psychological stress model in which rats were exposed to chronic fear by using an improved communication box widely adopted by scholars (Junlin et al., 2018; Liping et al., 2018). To prove the scientificity and rationality of the model, we evaluated the model in terms of diet, behavior, neurotransmitters, and glucocorticoid receptors. Specifically, decreased daily food and water intake, lost weight, and decreased open-field horizontal motion of the model rats indicated an apparent state of depression (Hale et al., 2006; Nicholson, 2010). Additionally, serum ACTH and NE levels in the amygdala, hippocampus, and cortex increased remarkably, further supporting our behavioral analysis (Chapman and Stern, 2010; Gresch et al., 2010). High expression levels of the NR3C1 and NR3C2 glucocorticoid receptors in the cortex indicated that rats in the model group experienced a high-stress state (Zucchi et al., 2010). These data systematically demonstrated the successful establishment of a psychological stress model from the perspective of emotional phenotype and neurotransmitter and glucocorticoid receptor levels, ensuring the scientificity of the experiment.

### Imbalanced Gut Microbiota Under Psychological Stress

We found that real psychological stress alone had significant effects on the composition and diversity of gut microbiota in rats. There was no significant difference between the two groups at the phylum level: Bacteroidetes, Firmicutes, and Proteobacteria represented approximately 98% of the total microbiota, consistent with previous studies (Tremaroli and Backhed, 2012; Partrick et al., 2018). However, at the generic level, we found that the gut microbiota composition of the model rats differed dramatically from that of the control subjects, characterized by 19 genera with increased abundance and 17 genera with decreased abundance. Intriguingly, 11 genera related to psychological stress were identified, which haven’t been reported. The mechanism of stress affecting intestinal microbial composition is unclear, but has been reported to be caused by changes in intestinal motility and mucin secretion leading to alterations in the internal environment in which microorganisms live (Freestone et al., 2008; Bailey et al., 2011). Catecholamines, including NE, can alter the gene expression of some bacteria, leading to growth of certain communities. Lyte et al. (2011) found that stress-induced elevation of NE concentration in the intestinal tract led to changes in the gut microbiota of rats. Elevation of NE concentration was also detected in this study, and could be one mechanism linking stress and the alteration of gut microbiota.

Furthermore, the α-diversity of gut microbiota in the model group was significantly higher than that in the control group after long-term chronic psychological stress, which was different from the decreased gut microbiota diversity triggered by the CUMS (Sun et al., 2018), social disruption stressor (Bharwani et al., 2016), and social conflict stress (Partrick et al., 2018) models reported in other studies. In this study, diet, environment, and other aspects were consistent between the control group and model group, so the increased diversity was perhaps related to the application of the communication box for modeling, which eliminated the interference of physiological factors. Based on the above, our study provides new evidence that stress can induce gut microbiota disorders, although there will be some variability according to the model and experimental conditions used. Therefore, the characteristics of each stressor should be assessed based on the main effects on the organism. The effects of psychological stress and physiological stress on gut microbiota should be further studied to elaborate their differences.

### Correlation of Defective Intestinal and Blood–Brain Barriers and Gut Microbiota

Previous scholars have proved that the brain and intestine can communicate with each other through the vagus nerve, and neuroendocrine, immune, and metabolic pathways (Mayer, 2011; Foster and Mcvey Neufeld, 2013; De et al., 2014). In the presence of psychological stress, neurotransmitters, cytokines, and other components produced during bacterial translocation can affect the body’s mood by activating the nervous system or directly acting on the brain (Berthoud, 2010; Bercik et al., 2011; Cryan and O’Mahony, 2011). The intestinal barrier and BBB are key pathways of substance transfer between the intestine and brain, with the tight junction between intestinal mucosal epithelial cells and vascular endothelial cells playing a significant role (Groschwitz and Hogan, 2009). In this study, we found that expression levels of the tight junction proteins claudin5, occludin, α-actin, and ZO-1 in the amygdala, hippocampus, duodenum, jejunum, and ileum were decreased under psychological stress, which were highly related with the disordered gut microbiota when using correlation thermography. Five core microfloral taxa related to permeability were represented in Figure 5, which could play a leading role during bacterial translocation.

**FIGURE 6:**
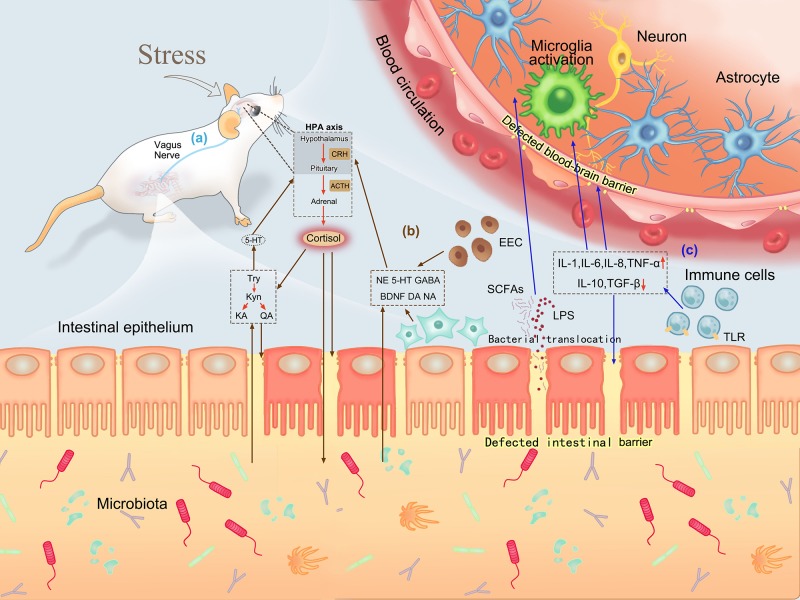
Roles of defective intestinal barrier and BBB in brain–gut communication. **(a)** Bidirectional interaction between the intestine and brain via the vagus nerve. **(b)** Neuroendocrine pathways in brain–gut communication. Psychological stress can activate the HPA axis and release cortisol. Cortisol can increase permeability by directly acting on intestinal mucosa or promoting tryptophan production, and change bacterial composition by influencing the intestinal environment. Increased intestinal permeability induces neurotransmitters and tryptophan-related products produced by intestinal endocrine cells and neurons to enter the blood circulation and react with the brain. **(c)** Inflammation and immune pathways in brain–gut communication. Psychological stress stimulates inflammatory responses and activates immune cells to release cytokines, which can destroy the integrity of the intestinal barrier and BBB. A defective intestinal barrier promotes more bacterial translocation, allowing bacterial metabolites to act on the brain through the defective BBB. Moreover, cytokines produced during immune activation can also stimulate microglia activation to affect mood.

It was a pity that we didn’t evaluate the changes in immune or metabolic substances in intestine or blood circulation. Nevertheless, based on the previous research reports, we could conclude the significant role that the defective intestinal and blood–brain barriers plays in the communication between gut and brain. The barriers are vital for sympathetic motor function (Udit and Gautron, 2013), neuroendocrine (Lyte, 2013), inflammation and immune activity (Wekerle, 2018), and bacterial metabolites (Chi et al., 2017), as shown in Figure 6. These findings explain the association and essential role of psychological stress-induced changes in gut microbiota with increased intestinal barrier and BBB permeability in the bidirectional interaction between gut microbiota and psychological stress, which indicate the occurrence and development of psychological diseases.

We hypothesize that defections of the intestinal barrier and BBB at multiple sites are essential links in brain–gut communication under psychological stress. Our findings provide a favorable research basis and direction for future study of the brain–gut axis and the pathogenesis of psychological diseases: whether the occurrence and development of psychological diseases can be blocked or treated by changing the permeability of the BBB and intestinal barrier.

Nevertheless, this study included very limited animals to study the correlation between gut microbiome and tight junction proteins of the intestinal and blood–brain barriers under psychological stress. Experiments employing a larger sample size are expected and urgently needed to verify the association. Also, we call for further research to elucidate the pathways in which stress affects such permeability and the material basis for mediating brain–gut communication, thus laying the foundation for the prevention and treatment of psychological disorders.

## Data Availability Statement

Sequencing data have been deposited under the number SRP201262 or PRJNA548591 in the NCBI database.

## Ethics Statement

The animal study was reviewed and approved by the Animal Ethics Committee of Henan University of Chinese Medicine.

## Author Contributions

SG performed the experiments and wrote the manuscript. LY provided the financial support, guided the design of experiments, and revised the manuscript. FC, ZZ, JL, WL, LC, YC, YB, ZF, YLin, ZL, SZ, HeW, and QZ carried out the experiments. YLi, XL, and JH guided the experiments. FC drew the diagram of the modeling device and the mechanism of brain-intestinal communication. JL and LC carried out the data analysis. HoW, XW, and JZ provided help in collecting literature.

## Conflict of Interest

The authors declare that the research was conducted in the absence of any commercial or financial relationships that could be construed as a potential conflict of interest.

## Footnotes

1 www.i-sanger.com
