# Supplementary material for: Gut Microbiota Are Associated With Psychological Stress-Induced Defections in Intestinal and Blood–Brain Barriers
Source: Front Microbiol. 2020 Jan 15;10:3067. doi: 10.3389/fmicb.2019.03067 (PMC6974438; doi:10.3389/fmicb.2019.03067)
Supplement: Supplementary file 1 [file Table_1.DOCX]

**Supplementary material**

**Table S1.** Comparison of α-diversity indexes between control and model groups at the genus level.

| Estimator | Control mean | Control SD | Model mean | Model SD | P-value |
| --- | --- | --- | --- | --- | --- |
| qstat | 16.938 | 1.6321 | 19.703 | 1.3099 | 0.008922 |
| bootstrap | 120.9 | 5.9098 | 130.33 | 5.3149 | 0.01569 |
| Sobs | 5.5 | 5.5767 | 123.5 | 4.4159 | 0.02031 |
| Ace | 122.83 | 6.7821 | 134.85 | 8.2629 | 0.02036 |
| jackknife | 127.51 | 7.6188 | 143.3 | 13.801 | 0.03402 |
| Chao | 125.26 | 8.9029 | 139.6 | 12.301 | 0.04329 |

Note: Multigroup comparison strategy: Student's t-test.
